# Tuning the spin coherence time of Cu(II)−(bis)oxamato and Cu(II)−(bis)oxamidato complexes by advanced ESR pulse protocols

**DOI:** 10.3762/bjnano.8.96

**Published:** 2017-04-27

**Authors:** Ruslan Zaripov, Evgeniya Vavilova, Iskander Khairuzhdinov, Kev Salikhov, Violeta Voronkova, Mohammad A Abdulmalic, Francois E Meva, Saddam Weheabby, Tobias Rüffer, Bernd Büchner, Vladislav Kataev

**Affiliations:** 1 Kazan E. K. Zavoisky Physical -Technical Institute, Russian Academy of Sciences, 420029 Kazan, Russia; 2 Technische Universität Chemnitz, Fakultät für Naturwissenschaften, Institut für Chemie, Straße der Nationen 62, D-09111 Chemnitz, Germany; 3 Department of Pharmaceutical Sciences, Faculty of Medicine and Pharmaceutical Sciences, University of Douala, BP 2701, Cameroon; 4 Leibniz Institute for Solid State and Materials Research IFW Dresden, D-01171 Dresden, Germany; 5 Institut für Festkörperphysik, Technische Universität Dresden, D-01062 Dresden, Germany

**Keywords:** electron spin echo, ESR, hyperfine interaction, molecular complexes, spin coherence

## Abstract

We have investigated with the pulsed ESR technique at X- and Q-band frequencies the coherence and relaxation of Cu spins *S* = 1/2 in single crystals of diamagnetically diluted mononuclear [*n*-Bu_4_N]_2_[Cu(opba)] (1%) in the host lattice of [*n*-Bu_4_N]_2_[Ni(opba)] (99%, opba = *o*-phenylenebis(oxamato)) and of diamagnetically diluted mononuclear [*n*-Bu_4_N]_2_[Cu(opbo*n*-Pr_2_)] (1%) in the host lattice of [*n*-Bu_4_N]_2_[Ni(opbo*n*-Pr_2_)] (99%, opbo*n*-Pr_2_ = *o*-phenylenebis(*N*(propyl)oxamidato)). For that we have measured the electron spin dephasing time *T*_m_ at different temperatures with the two-pulse primary echo and with the special Carr–Purcell–Meiboom–Gill (CPMG) multiple microwave pulse sequence. Application of the CPMG protocol has led to a substantial increase of the spin coherence lifetime in both complexes as compared to the primary echo results. It shows the efficiency of the suppression of the electron spin decoherence channel in the studied complexes arising due to spectral diffusion induced by a random modulation of the hyperfine interaction with the nuclear spins. We argue that this method can be used as a test for the relevance of the spectral diffusion for the electron spin decoherence. Our results have revealed a prominent role of the opba^4–^ and opbo*n*-Pr_2_^4–^ ligands for the dephasing of the Cu spins. The presence of additional ^14^N nuclei and protons in [Cu(opbo*n*-Pr_2_)]^2–^ as compared to [Cu(opba)]^2–^ yields significantly shorter *T*_m_ times. Such a detrimental effect of the opbo*n*-Pr_2_^4−^ ligands has to be considered when discussing a potential application of the Cu(II)−(bis)oxamato and Cu(II)−(bis)oxamidato complexes as building blocks of more complex molecular structures in prototype spintronic devices. Furthermore, in our work we propose an improved CPMG pulse protocol that enables elimination of unwanted echoes that inevitably appear in the case of inhomogeneously broadened ESR spectra due to the selective excitation of electron spins.

## Introduction

Cu(II)−(bis)oxamato and Cu(II)−(bis)oxamidato complexes have attracted in the recent past substantial attention as precursor materials for the synthesis of the corresponding polynuclear complexes which in their turn have been investigated with regard to the magnetic superexchange interactions between the Cu spins mediated by the O and N ligands [1–8]. In this context, the transfer of the spin density from the central metal ion to the ligands and next via the oxamato or oxamidato unit bridging two neighbored paramagnetic transition metal ions is important for the maintaining of the superexchange interaction. This transfer also gives rise to the hyperfine (HF) coupling between the Cu electron spin *S* = 1/2 and ^14^N nuclear spins *I* = 1 which has been studied with ESR techniques in some detail [8–11]. On the other hand, the dynamics of electron spins, the spin coherence and spin relaxation processes in such complexes have been scarcely addressed so far. Such knowledge is however equally important from the fundamental point of view and also by considering possible applications of mono- and polymetallic Cu(II)−(bis)oxamato and Cu(II)−(bis)oxamidato complexes in molecular electronic devices.

Pulse methods of electron spin resonance (ESR) have been shown to be very informative in assessing the magnetically active molecular complexes for the purpose of quantum information processing. With these techniques, one can directly measure the electron spin coherence times and, moreover, can manipulate the spin states in order to perform quantum logical operations [12–23]. For measurements of the electron spin dephasing time *T*_m_ most commonly the simple primary Hahn echo method employing two pulses that rotate the spins at resonance by 90° (π/2) and 180° (π) was used: π/2 – τ – π – τ – echo. Recently we have shown that the application of a more sophisticated, so-called Carr–Purcell–Meiboom–Gill (CPMG) multiple microwave pulse sequence [24–25] can boost the *T*_m_ time in molecular complexes up to one order of magnitude [20]. The CPMG pulse protocol can efficiently reduce the manifestation of the unwanted decoherence channel, referred to as spectral diffusion, that arises due to the random modulation of the HF interaction of electron spins with surrounding nuclear spins. It should be noted that the slowing down of the spin decoherence in the multi-pulse CPMG experiments is of special interest with regard to quantum computation on molecular electron spins (see, e.g., [26]), since the realization of the logical operations requires special pulse sequences. For example, it has been shown in [27] that for the realization of the quantum logical operation CNOT on two electron spins it is necessary to apply about twenty microwave pulses. Thus, it is obviously important to take into account the influence of the multiple pulse protocols on the decoherence of spins on which the quantum logical operations are performed.

**Figure 1:**
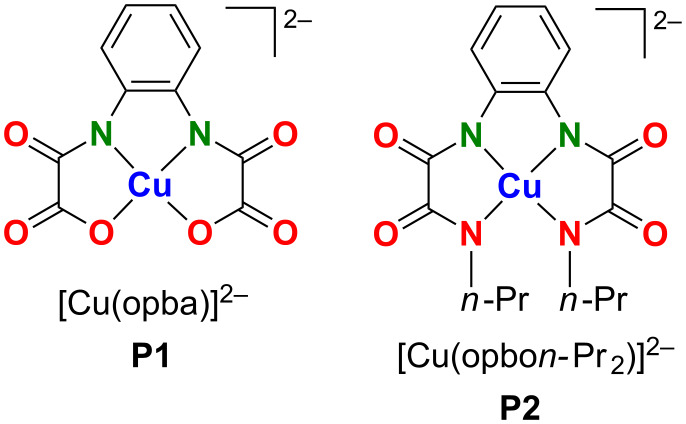
Chemical structures of the anionic complex fragments [Cu(opba)]^2−^ (**P1**, left) and [Cu(opbo*n*-Pr_2_)]^2−^ (**P2**, right).

In the present work, we have investigated the temperature and magnetic field/frequency dependence of the spin dephasing time *T*_m_ in the single-crystalline samples of Cu(II)−(bis)oxamato and Cu(II)−(bis)oxamidato molecular complexes with pulse ESR at the X- and Q-band frequencies. The first complex is the diamagnetically diluted mononuclear [*n*-Bu_4_N]_2_[Cu(opba)] complex (1%) in the host lattice of [*n*-Bu_4_N]_2_[Ni(opba)] (99%, opba = *o*-phenylenebis(oxamato)), and the second one is the diamagnetically diluted mononuclear [*n*-Bu_4_N]_2_[Cu(opbo*n*-Pr_2_)] complex (1%) in the host lattice of [*n*-Bu_4_N]_2_[Ni(opbo*n*-Pr_2_)] (99%, opbo*n*-Pr_2_ = *o*-phenylenebis(*N*(propyl)oxamidato)) (Figure 1). We have shown that the CPMG pulse sequence can maintain the spin coherence on the time scale of up to ≈10 µs at low temperatures in the first complex whereas the spin dephasing in the second complex occurs on a shorter time scale. We relate this difference with the detrimental influence of the HF interaction with additional ^14^N nuclei and protons in the Cu(II)−(bis)oxamidato complex whereas this unwanted effect is reduced in the Cu(II)−(bis)oxamato complex containing less ^14^N nuclei and protons. In fact, a multi-pulse CPMG sequence which can slow down the phase relaxation of electron spins as compared to the primary echo, the effect which we earlier suggested to be a manifestation of the quantum Zeno effect in multi-pulse experiments [20], can be proposed as a method to reveal on a phenomenological level the contribution of the spectral diffusion to the electron spin phase relaxation. Furthermore, in our experiments we were confronted with the situation that required a modification of the CPMG pulse protocol. The common CPMG theory assumes that all pulses in the sequence unselectively rotate all spins by the same angle of π/2 for the first pulse and of π for the other pulses. However, often in reality the spins are rotated selectively so that the different sub-ensembles of isochromatic spins are turned by the microwave pulses by different angles, yielding additional echoes such as the stimulated echo. Indeed, by applying the standard CPMG pulse protocol we have observed that the CPMG echoes are distorted by additional echoes arising due to the selective excitation of electron spins by the pulses. We have proposed a modified CPMG pulse protocol, applying which we could successfully eliminate these contributions.

## Experimental Results

In this work two single-crystalline samples of [*n*-Bu_4_N]_2_[Cu(opba)] and [*n*-Bu_4_N]_2_[Cu(opbo*n*-Pr_2_)], denoted in the following as **P1** and **P2** (Figure 1), diamagnetically diluted in the host lattices of their corresponding and diamagnetic Ni(II)-containing complexes [*n*-Bu_4_N]_2_[Ni(opba)] and [*n*-Bu_4_N]_2_[Ni(opbo*n*-Pr_2_)] have been studied with pulse ESR techniques at X- and Q-band microwave frequencies of ≈9.8 GHz and 33.9 GHz, respectively. The accessibility of such diamagnetically diluted single crystals has been recently described by some of us [10,28]. Due to the coordination of two deprotonated amido nitrogen atoms and two carboxylate oxygen atoms for **P1** the formation of a CuO_2_N_2_ coordination unit was observed [28], cf. Figure 1. It is to note, that not only the CuO_2_N_2_ unit but the whole complex fragment **P1** was observed as nearly ideally planar [28]. Moreover, the complex fragment **P1** in the diamagnetically diluted single crystals described here is expected to have crystallographically imposed *C*_2_ symmetry, as [*n*-Bu_4_N]_2_[Cu(opba)] and [*n*-Bu_4_N]_2_[Ni(opba)] are structurally isomorphic and possess *C*_2_ symmetric complex fragments as well [28]. For **P2**, due to the coordination of four deprotonated amino nitrogen atoms, the formation of a CuN_4_ coordination unit was observed [10], cf. Figure 1. Compared to **P1**, deviations from planarity of the CuN_4_ unit and of **P2** are significantly larger [10]. Furthermore, **P2** should be *C*_1_ symmetric in the here described diamagnetically diluted single crystals, cf. crystallographic data and descriptions in [10].

### Measurement details

ESR measurements were performed with an Elexsys E580 spectrometer from Bruker operating at X- and Q-bands. The spectrometer is equipped with the standard cavities (ER4118MD5-W1 for X-band and EN5107D2 for Q-band measurements). For the temperature dependent measurements the cavities are inserted into the CF935 cryostat. The temperature is controlled with the ITC503 temperature controller from Oxford Ins.

Echo-detected ESR spectra were recorded by using the standard primary echo method with the subsequent integration of the echo signal during the magnetic field sweep at each field point.

For the measurements of the phase memory time *T*_m_ two pulse protocols were used: the primary echo decay and the decay of the Carr–Purcell–Meiboom–Gill echoes [24–25]. With the first protocol, π/2 – τ – π – echo, the echo intensity was measured as a function of the time interval τ. The initial τ value of 200 ns was always used, and the increment of τ amounted to 20 ns for both X- and Q-band measurements. The length of the π/2-pulse amounted to 100 ns and 26 ns for X- and Q-bands, respectively. The long selective π/2-pulse of 100 ns was used to avoid the modulation of the echo envelope due to the interaction of electron spins with protons (the so-called ESEEM effect) [29–31].

In the CPMG sequence (π/2)_x_ – { τ − (π)_y_ − τ − echo −}*^n^*- the length of the π/2-pulse was set to 16 ns and 26 ns at X- and Q-bands, respectively. Due to technical limitations the delay time τ cannot be set shorter as 300 ns. To enable measurements of both complexes in a broad temperature range and at two frequencies with the same value of τ, we have fixed it at 400 ns. The number *n* of the π-pulses was chosen such, so that the last *n*-th echo could not be observed anymore above the noise level. For example, at *T* = 10 K it was possible to apply 250 π-pulses, whereas the number of echoes reduced down to *n* = 25 at 80 K. To evaluate the longitudinal relaxation time the stimulated echo (SE) decay was measured at the Q-band. The SE pulse protocol reads: π/2 – τ – π/2 – *t* – π/2 – τ – SE. With this protocol, the SE intensity is measured as a function of time *t*. The length of the π/2-pulse amounted to 20 ns, the τ value was equal to 200 ns, and the initial value of *t* started from 800 ns.

### X-band results

**Figure 2:**
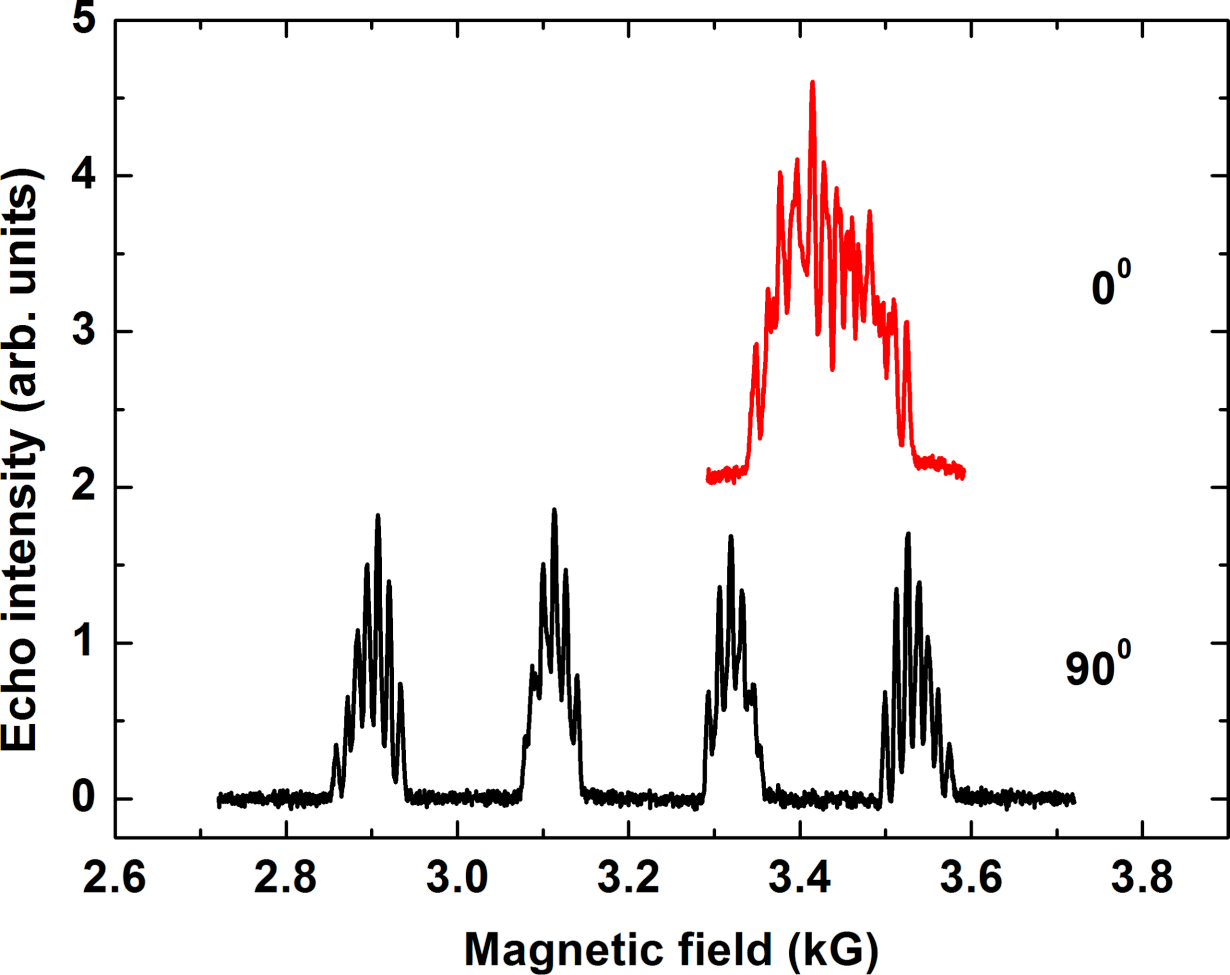
Echo detected ESR spectra of **P1** at a frequency ν = 9.85 GHz (X-band) and at *T* = 20 K for the magnetic field oriented normal (90°) and parallel (0°) to the molecular plane.

Representative spin echo detected ESR spectra of **P1** are shown in Figure 2 for two orientations of the magnetic field ***H***. For ***H*** normal to the molecular plane (***H***||*z*-axis) the spectrum consists of four groups of lines arising due to the on-site hyperfine (HF) interaction between the Cu spin *S* = 1/2 with the ^63,65^Cu nuclear spins *I* = 3/2. Each group is further structured due to the HF-coupling with the ^14^N nuclear spins *I* = 1 of the two N ligands (Figure 2). For the in-plane orientation (***H***||*xy*-plane) this group of lines collapses into an only partially resolved spectrum. Similar ESR spectra, though with the less resolved ^14^N HF structure due to the presence of four instead of two N-donor ligands, were obtained for **P2** (not shown).

Measurements of the phase relaxation time *T*_m_ of both complexes with the primary echo and CPMG pulse sequences have revealed that *T*_m_ does not depend on the choice of the peak of the ESR spectrum where the pulse sequences were applied and on the orientation of the field.

**Figure 3:**
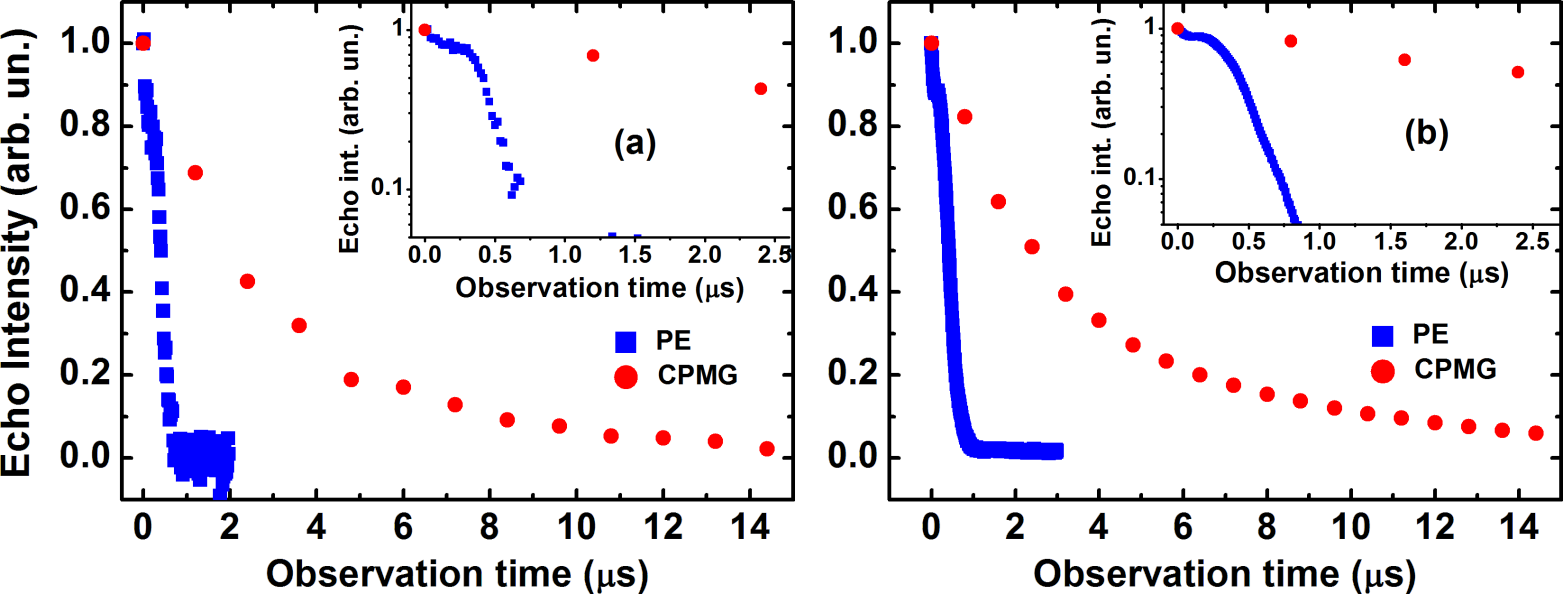
Time dependence of the intensity of the echo signal for complex **P1** at *T* = 30 K on a linear (main panel) and on a logarithmic scale (inset) measured with the primary echo (blue squares) and CPMG (red circles) protocols at X-band (a) and at Q-band (b).

Typical time dependences of the spin echo decay for complex **P1** obtained with the primary echo and CPMG pulse sequences are shown in Figure 3a. The primary echo decay is modulated for both, X- and Q-band, measurements (Figure 3), and the modulation frequency is rather low ≈0.3 MHz. We suppose that this modulation effect occurs due to the non-secular part of a type *S*_z_*I*_x_ of the HF interaction between the Cu electron spin and the nitrogen nuclei [32]. Note that this particular part of the HF interaction is responsible for the excitation of the forbidden transitions induced by the microwave pulses which produce the electron spin echo signal, so that allowed and forbidden ESR transitions manifest the coherence in their excitation by the microwave pulses. This spin coherence is manifested as the ESEEM effect [29–31].

**Figure 4:**
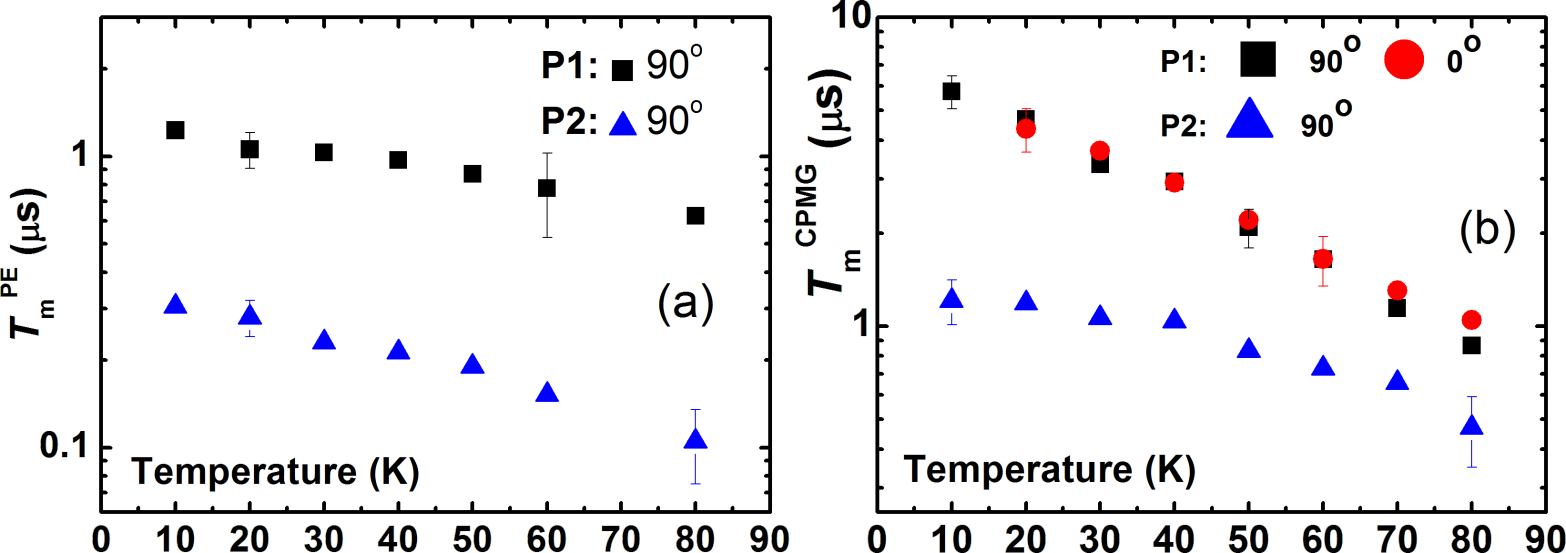
Temperature dependence of the phase relaxation time *T*_m_ of **P1** and **P2** at a frequency ν = 9.85 GHz measured with the primary echo sequence for the 90° field orientation (a) and with the CPMG pulse sequence for 0° and 90° orientations of the magnetic field (b).

The spin echo decay curves were fitted with the stretched exponential function *y* = *y*_0_ + *A·*exp(−2τ/*T*_m_)*^b^*, where *b* is the exponent index characterizing the spread of the relaxation times. For **P1**, the fit reveals *b* ≈ 2 for the primary echo decay indicating the effect of the spectral diffusion [33–35], whereas a smaller value of the exponent *b* ≈ 0.8 − 1 characterizes the primary echo decay of **P2**. The exponent *b* ≈ 1 was found for the decay of the CPMG echoes for both complexes revealing their mono-exponential character. Representative *T*-dependences of *T*_m_ for **P1** and **P2** are shown in Figure 4. Evidently, an application of the CPMG pulse protocol leads to a significant enhancement of the *T*_m_ time, by a factor of ≈6 and ≈4 at the lowest temperature for complexes **P1** and **P2**, respectively. On the absolute scale, however, the spin decoherence of **P2** is sigificantly faster as compared to **P1** regardelss the applied pulse sequence.

### Q-band results

**Figure 5:**
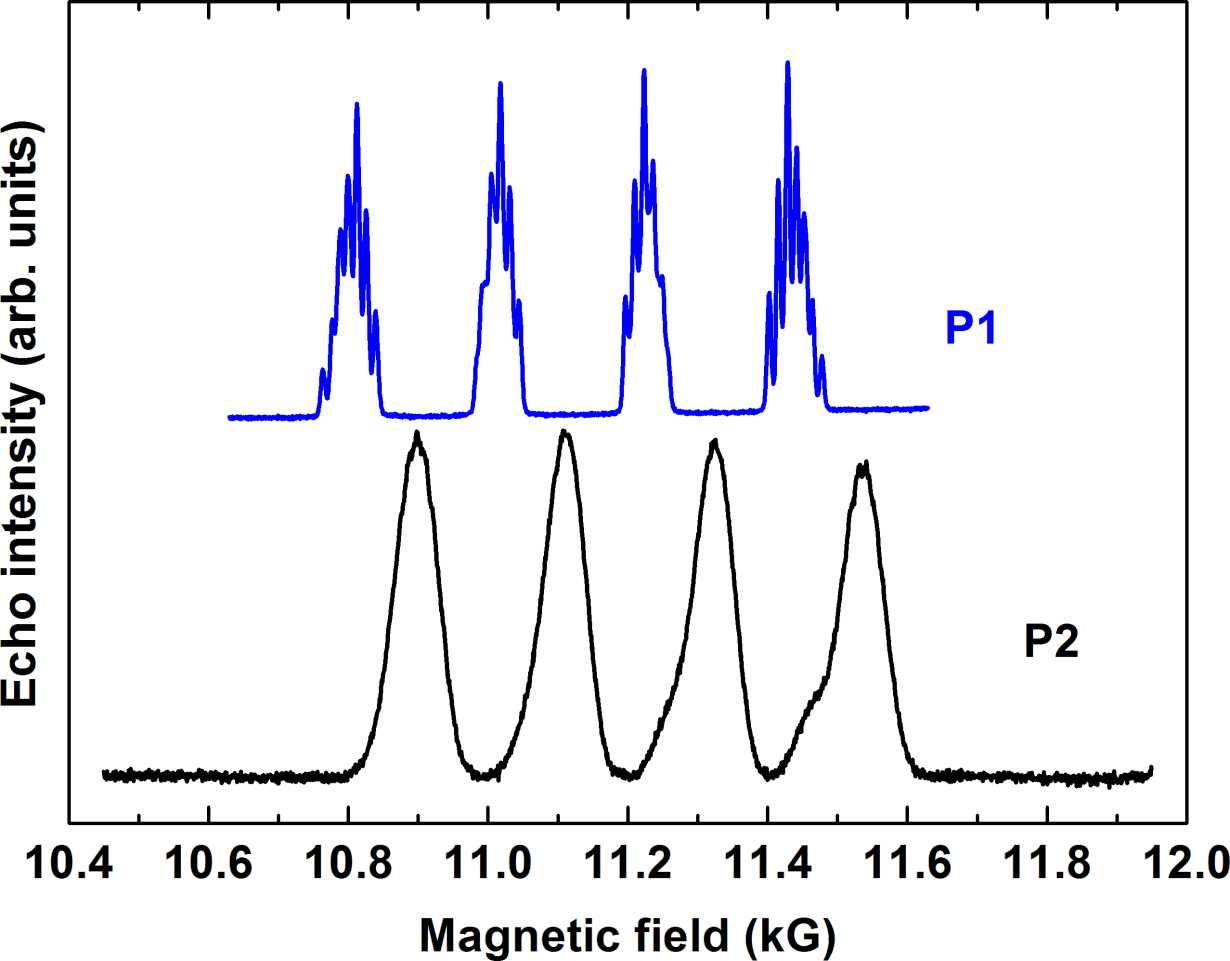
Echo detected ESR spectra of **P1** (top) and **P2** (bottom) at a frequency ν = 33.899 GHz (P1) and 33.915 GHz (P2) (Q-band) and at *T* = 20 K for the magnetic field oriented normal (90°) to the molecular plane. The difference of the line positions of the two spectra is due to the different *g*-factor of Cu for the studied samples *g*_z_(P1) = 2.184 [9] and *g*_z_(P2) = 2.159 [10].

Echo detected ESR spectra of complexes **P1** and **P2** in the Q-band frequency range have revealed similar features as the X-band spectra. The quartet group of peaks due to the on-site HF coupling with the ^63,65^Cu nuclear spins is most extended for the magnetic field applied normal to the molecular plane (90° orientation). The structure of each peak due to the HF-coupling with the ^14^N nuclear spins is visible only for complex **P1** with two N-donor ligands only. Representative spectra for ***H***||*z*-axis are shown in Figure 5. The small shoulders visible at the two high-field peaks in the spectrum of **P2** are presumably related to a small amount of powder inclusions in the sample. It appears that unavoidable thermal cycling of the sample between 10 K and room temperature during the experiments yields microcracks and partial crumbling of some parts of the crystal.

**Figure 6:**
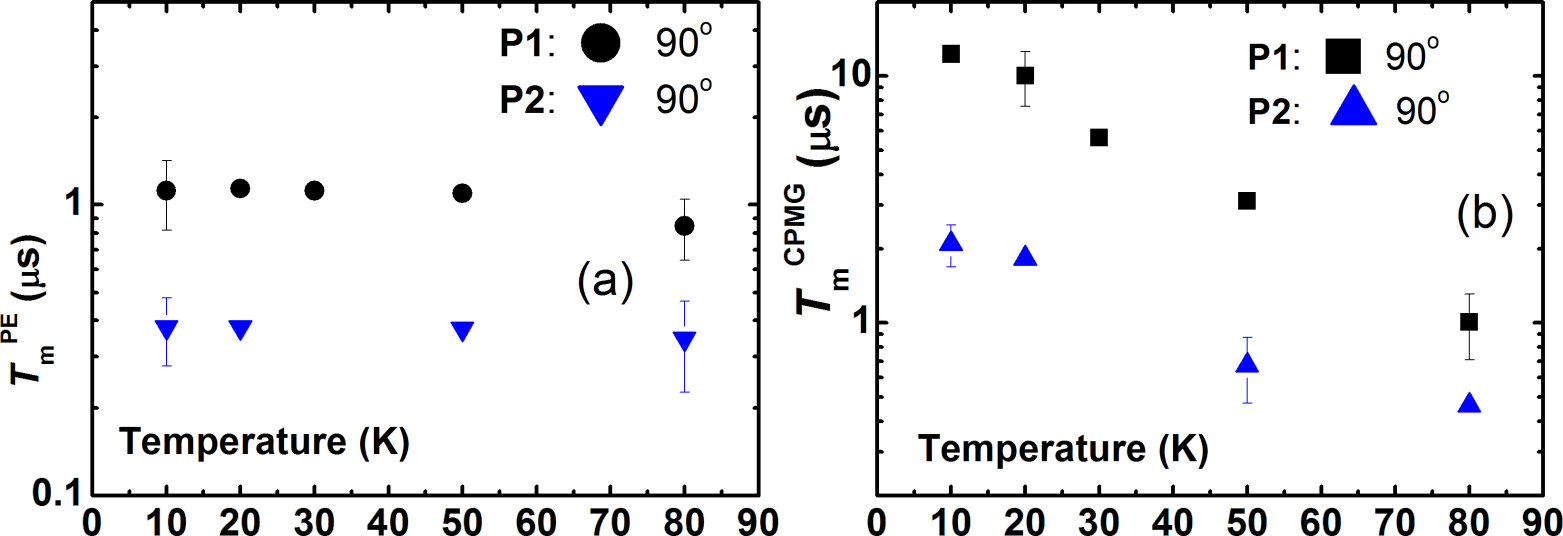
Temperature dependence of the phase relaxation time *T*_m_ of **P1** and **P2** at a frequency ν = 33.9 GHz for the 90° field orientation measured with the primary echo sequence (a) and with the CPMG pulse sequence (b).

Similar to the X-band results, the *T*_m_ spin dephasing time for both complexes did not depend on the magnetic field orientation and on the choice of the spectral peak where the measurement took place. Typical spin echo decay curves are shown in Figure 3b. Temperature dependences of *T*_m_ measured with the primary echo and CPMG pulse sequences are presented in Figure 6.

The spin coherence time *T*_m_^CPMG^ obtained with the CPMG pulse protocol is enhanced by a factor of ≈2 at the lowest temperature as compared to the *T*_m_^CPMG^ time in the X-band measurements.

**Figure 7:**
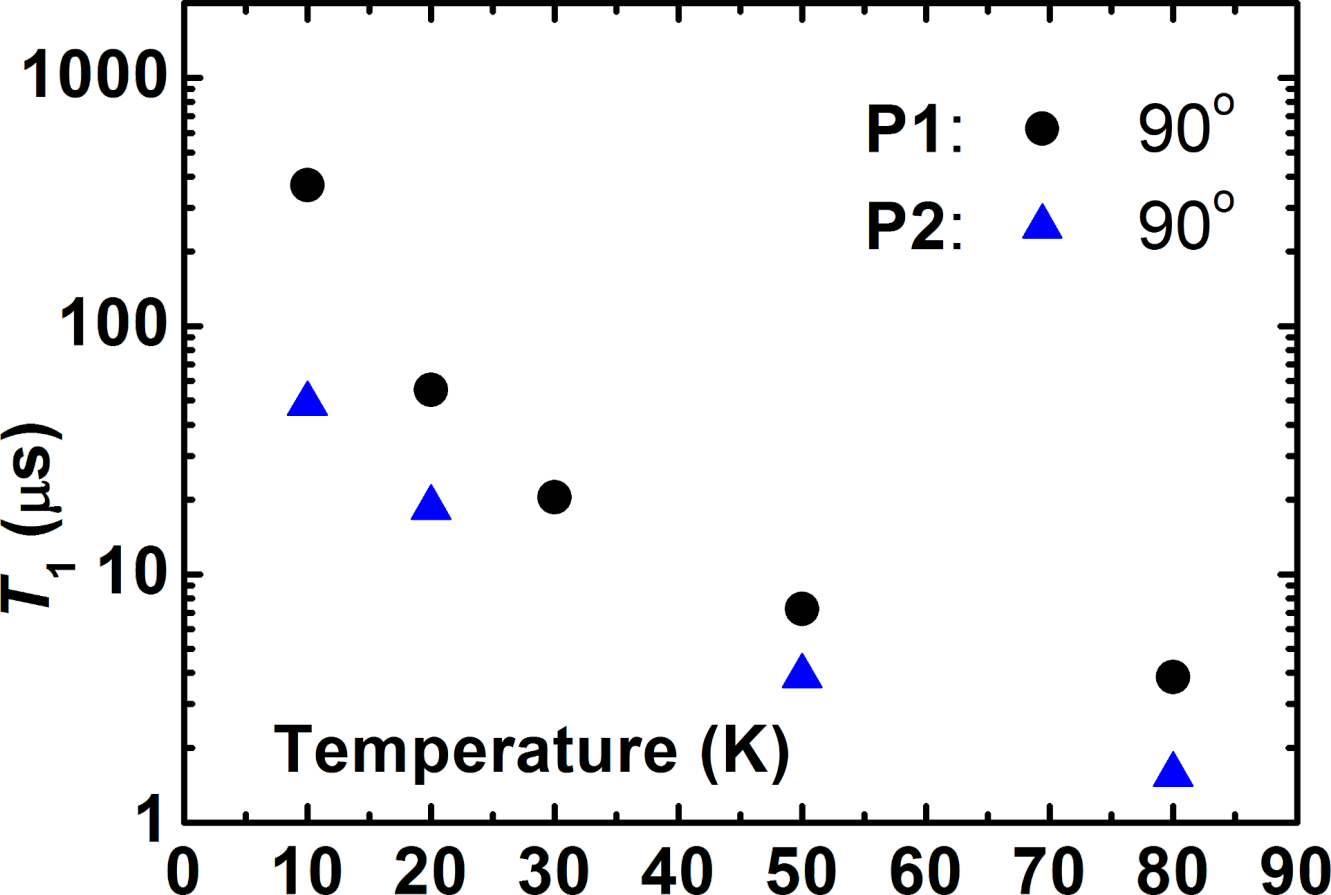
Temperature dependence of the longitudinal relaxation time *T*_1_ of **P1** and **P2** at a frequency ν = 33.9 GHz for the 90° field orientation.

Finally, to ensure that the longitudinal relaxation does not influence the electron spin decoherence, the *T*_1_ time was measured for both complexes. As can be seen in Figure 7, at all studied temperatures *T*_1_ is always longer than the respective *T*_m_ times, cf. Figure 4 and Figure 6.

### Peculiarities of the CPMG spin echoes

**Figure 8:**
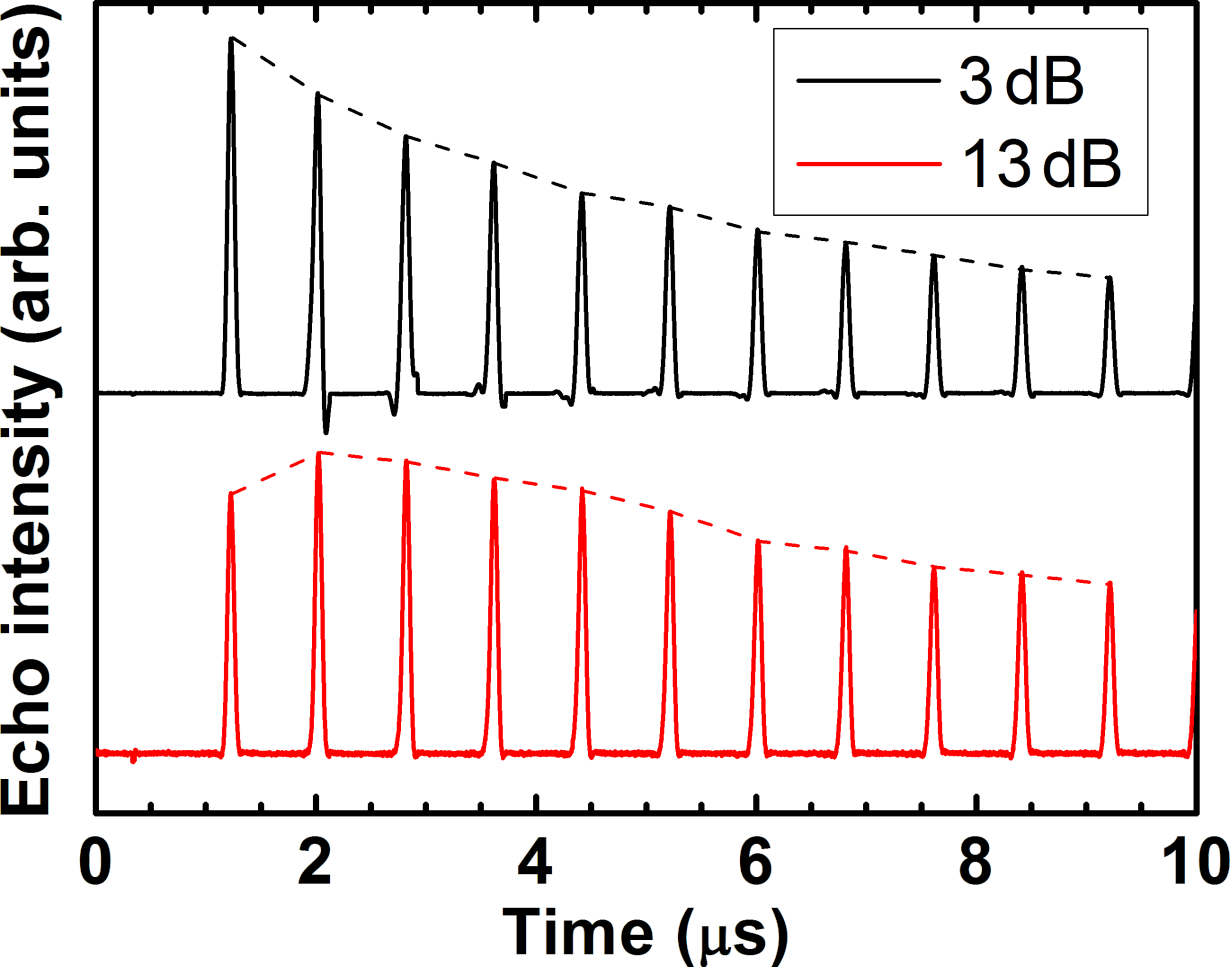
CPMG echoes for complex **P1** for two levels of the microwave power attenuation of 3 dB and 13 dB. Note that in the latter case the 2nd and not the 1st echo has the strongest amplitude.

**Figure 9:**
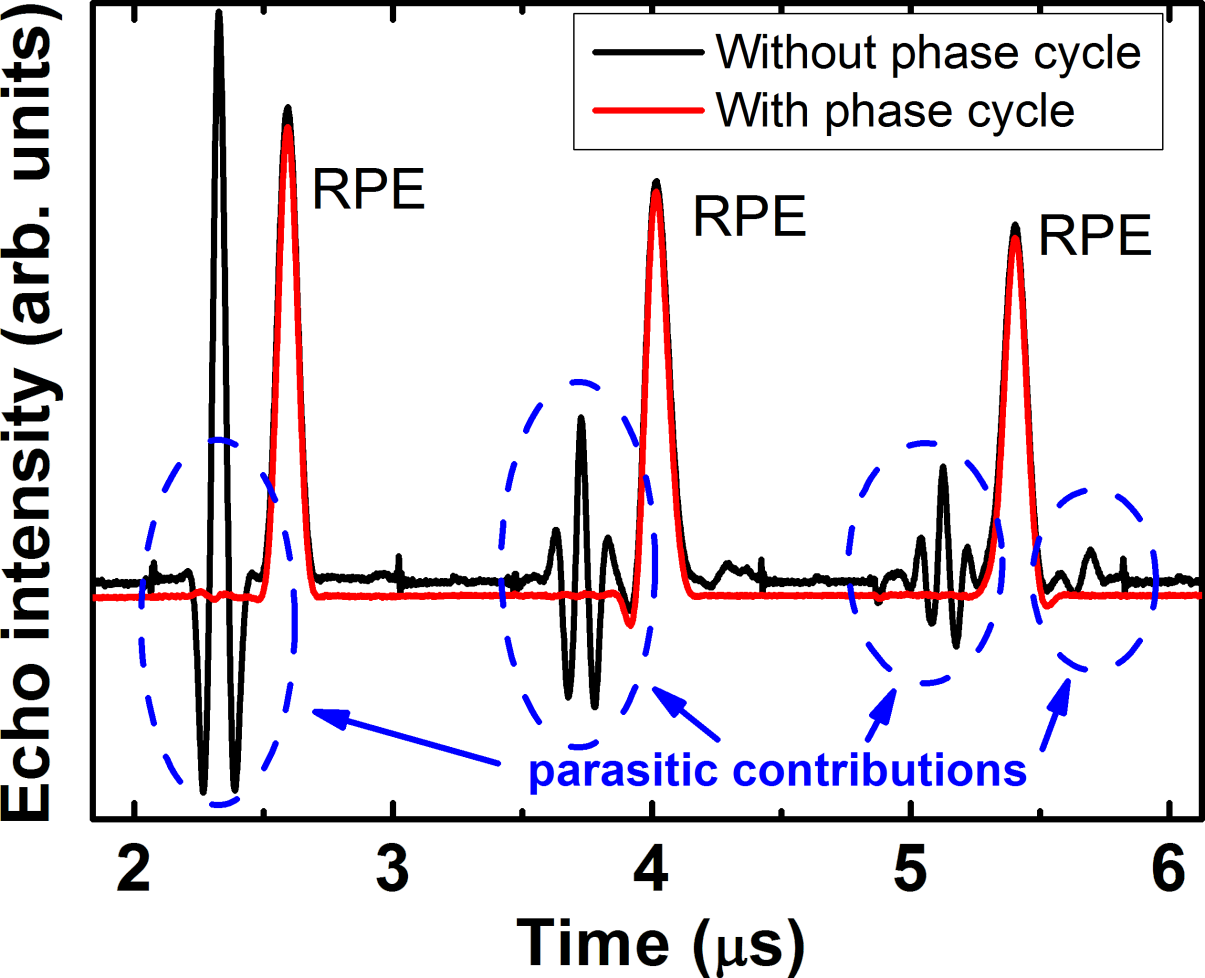
CPMG experiment on complex **P1** at ν = 33.9 GHz, *T* = 20 K, and ***H***||*z*-axis: Separation of the refocused primary echoes (RPE) from other parasitic contributions by applying the modified CPMG pulse (π/2)_x_ – τ_1_ – (π)_y_ – τ_1_ – echo – {τ_2_ – (π)_y_ – τ_2_ – echo}^(^*^n^*^ − 1)^ with τ_1_ = 400 ns and τ_2_ = 700 ns (black line). The signal plotted in red is the result of the phase cycling of the first π pulse that effectively suppresses the “parasitic” contributions to the true CPMG echoes (see the text).

Application of the conventional CPMG pulse protocol (π/2)_x_ – *τ* – (π)_y_ – *τ* – echo – { *τ* – (π)_y_ – *τ* – echo}^(^*^n^*^ − 1)^ enhances the spin coherence lifetime of the studied complexes. In addition, the experiments have revealed a dependence of the CPMG echoes on the applied microwave power. At small power levels, the second echo appears larger in amplitude than the first primary echo (Figure 8). In the ideal CPMG experiment, each echo generated by the *n*-th π pulse is the *n*-th refocused primary echo (RPE) which amplitude decreases with *n* due to the inevitable spin decoherence. Thus, the observed nonmonotonous behavior suggests that in addition to the RPE other unwanted echoes contribute to the signal. To separate the RPE from those contributions, a modified pulse sequence (π/2)_x_ – τ_1_ – (π)_y_ – τ_1_ – echo – {τ_2_ – (π)_y_ – τ_2_ – echo}^(^*^n^*^ − 1)^ has been applied. As a result, the RPEs occur always at the time delay τ_2_ after the *n*-th π pulse, whereas, for instance the stimulated echo, which can occur due to the incomplete rotation by the pulses of the spins that are slightly off the resonance, has an offset from the RPE by |τ_2_–τ_1_| (Figure 9). In this way, the “parasitic” contributions to the true CPMG echoes can be identified and separated. Furthermore, they can be successfully eliminated by applying the phase cycling, following the general rules of the cycling of the phases of microwave pulses (see, e.g., [31,36]). In the first run, the first π pulse in the CPMG sequence is applied about the +*y*-axis, (π)_y_, whereas in the second run it is applied about the −*y*-axis (π)_−y_, and the two runs are summed up. As a result, an almost perfect sequence of RPEs has been obtained (Figure 9).

## Discussion

Our experiments on the molecular complexes **P1** and **P2** demonstrate clearly that the dephasing time of the Cu spins *S* = 1/2 can be significantly enhanced by the application of the CPMG pulse protocol. Analogous effect of a drastic increase of the spin coherence time in molecular magnets with the CPMG protocol was observed and comprehensively discussed in our previous work on pulse ESR on model binuclear 1,2-diphosphacyclopentadienyl manganese complexes [20]. There we have studied in detail the slowing down of the electron spin decoherence in a CPMG experiment if the decoherence is caused by the stochastic process of the spectral diffusion. In that our work [20] we have interpreted such slowing down as a manifestation of the quantum Zeno effect in multi-pulse experiments. Thus the occurrence of this effect signifies that the spin decoherence is related to some process of spectral diffusion.

Generally, electron spin decoherence can be due to spin-lattice interaction, spin-rotation interaction, HF interaction with magnetic nuclei as well as spin-spin interaction. On a specific example we considered in [20] the situation when stochastic changes of the resonance frequency of an electron spin are caused by stochastic modulation of the HF interaction of an electron with magnetic nuclei. Also in the present work, considering the above described experimental results on the magnetically diluted Cu(II)–(bis)oxamato and Cu(II)–(bis)oxamidato complexes, it is reasonable to conclude that the major contribution to the decoherence of the Cu(II) electron spins is given by the HF interaction with the nuclear spins of the host lattice.

Electron spin decoherence due to the HF interaction with surrounding nuclei has been studied in a number of works beginning with the pioneering work by Gordon and Bowers [37]. In [30,33,38] a detailed theoretical and experimental investigation of the kinetics of the decay of the envelope of the primary spin echo of hydrogen atoms in frozen solutions of H_2_SO_4_ and in fused quartz has been made. The shift of the ESR resonance frequency can be written in the form Δω = ∑*A_k_M_k_*, where *A_k_* and *M_k_* are the HF constant and the projection of the *k*-th nuclear spin on the direction of the external magnetic field. For a number of reasons the projection *M_k_* can stochastically change in time. This can be due to the spin-lattice relaxation. The parameters *A_k_* can also randomly change due to molecular motion, lateral and rotational diffusion. One expects that in solids at low temperatures the more effective mechanism of the spectral diffusion arises from the stochastic variations of *M_k_* due to the spin diffusion, i.e., the stochastic mutual flip-flops of two nuclear spins induced by the nuclear dipole–dipole interaction. For example, a characteristic flip-flop frequency of the neighboring protons in a lattice is of the order 10^4^–10^5^ 1/s, i.e., flip-flops occur on the time scale 10^−4^–10^−5^ s. Thus, the shift of the ESR frequency of the electron spin is a stochastic process ω(*t*). A stationary distribution of these frequencies determines the HF structure of an ESR spectrum. The theory in [30,33,38] shows that the primary electron spin echo signal decay resulting from the random modulation of the hyperfine interaction by nuclear spin diffusion obeys the expression

[1]
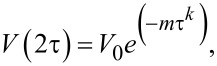


where 7/4 ≤ *k* ≤ 3. In the region of a comparatively small τ, *k* = 3, whereas for large τ the exponent *k* in Equation 1 takes the value *k* = 7/4.

For the nuclear spins *I* = 1/2 in the limit of large τ one has [30,33,38]:

[2]
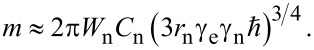


Here, *C*_n_ is the concentration of magnetic nuclei, γ_e_ and γ_n_ are the electron and nuclear gyromagnetic ratios, respectively, 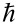 is the reduced Planck constant, *r*_n_ is the distance between the neighboring magnetic nuclei, and *W*_n_ is the nuclear flip-flop rate [39]:

[3]
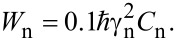


In fact, the parameter *m* depends on the diffusion barrier for nuclear spins in the vicinity of the unpaired electron. The dipolar magnetic field of the unpaired electron is differently shifting the resonance frequencies of the nuclear spins at different spatial positions. At close distances, the difference of the nuclear spins’ frequencies exceeds the strength of their mutual nuclear dipole–dipole interaction so that their mutual flip-flop process is inhibited. Thus, nuclear spin diffusion stops close to the unpaired electron, at a distance which is referred to as a diffusion barrier for nuclear spins. The radius of the spin diffusion barrier *d* is estimated to be about *d* ≈ 1 nm [38]. The parameter *m* (Equation 2) is expected to be larger if *d* is reduced. This point has to be kept in mind when studying the electron spin decoherence for different paramagnetic centers.

For complexes **P1** and **P2** studied in the present work we do not expect significantly different radii of the nuclear spin diffusion barrier. From Equation 2 and Equation 3 it follows that *m* ~ *C*_n_^2^. The concentration of protons and nitrogen nuclei in **P1** is smaller than in **P2** (Figure 1). Thus, from the above considerations, the electron spin decoherence rate in complex **P1** is expected to be smaller than in complex **P2** which agrees qualitatively very well with the experimental observations (Figure 3 and Figure 6).

Though the kinetics of the decay of the primary and stimulated echoes due to the spectral diffusion induced by the stochastic modulation of the HF interaction in the presence of the nuclear spin diffusion was theoretically elaborated in [38], the manifestation of this mechanism for a CPMG pulse protocol was first theoretically addressed in [20] in the framework of the model of a normal stochastic process. If the number of magnetic nuclei that effectively interact with an electron is sufficiently large (e.g., larger than 5), then in a good approximation the respective frequency distribution can be described by the Gaussian with the dispersion

[4]
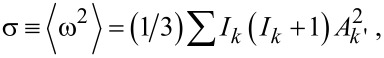


where *I_k_* is the spin of the *k*-th nucleus. It should be noted that the magnetic nuclei nearest to the electron, i.e., those located closer than the radius of the diffusion barrier *d*, do not contribute to the spectral diffusion. Therefore, in Equation 4 the dispersion σ is determined by the HF interaction with the nuclear spins at distances larger than *d*. Thus, in complexes **P1** and **P2** studied in the present work the HF interaction of the Cu(II) electron spin with its own ^63,65^Cu nucleus and with the ^14^N nuclei of the nearest ligands most likely do not contribute to the spectral diffusion whereas distant ^63,65^Cu and ^14^N nuclei can contribute to this process. This means that, unfortunately, the parameter σ in Equation 4 cannot be calculated simply as a dispersion of a usual continuous wave ESR spectrum that contains contribution of all magnetic nuclei, both within and outside the diffusion barrier sphere of the radius *d*.

For quantitative estimates, it is necessary to describe the stochastic process ω(*t*). In [20] a phenomenological model of the normal stochastic process has been considered for the description of the spectral diffusion. In this model such a process can be fully described by the dispersion of the frequency distribution and the frequency correlation function

[5]
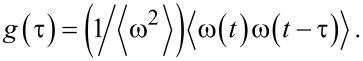


We can define a characteristic time of the decay of the frequency correlation as τ_c_, and assume that the correlation function *g*(τ) (Equation 5) has an exponential form:

[6]
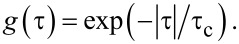


In the case of the spectral diffusion induced by the nuclear spin diffusion τ_c_ is of the order of the flip-flop time of the neighboring nuclear spins. If those spin-flops are caused by the nuclear dipole–dipole interaction, then τ_c_ ≈ 10^−4^–10^−5^ s [39]. According to [20], the expression for the *n*-th signal in a CPMG experiment in the presence of spectral diffusion reads:

[7]
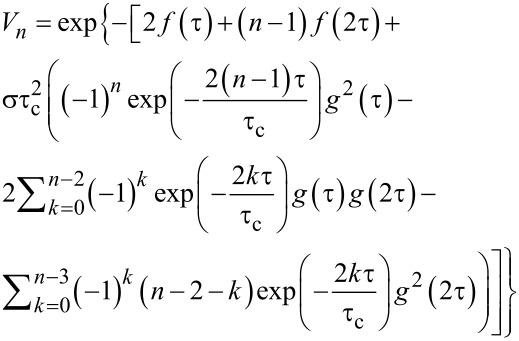


In Equation 7 the functions *f*(*t*) and *g*(*t*) are defined as:

[8]
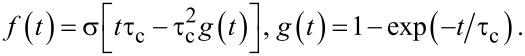


Setting *n* = 1 in Equation 7 yields the expression for the amplitude of the primary echo

[9]
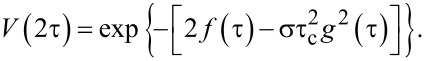


For short observation times τ << τ_c_, Equation 9 reduces to

[10]
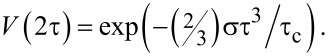


(Note that in [20] Equation 10 was mistakenly written as *V*(2τ) = exp(−2στ^2^), cf. Eq. (11) in [20]). For long observation times τ >> τ_c_, it follows from Equation 9 that the primary echo signal exponentially depends on τ:

[11]
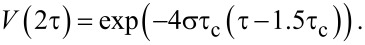


It can be concluded from Equation 10 and Equation 11 that in the framework of the phenomenological model of the normal stochastic process for the description of the spectral diffusion due to the stochastic modulation of the HF interaction by the nuclear spin diffusion, the dependence of the decay of the primary echo signal has the form exp(−*m*τ*^k^*) where the index *k* changes from 3 to 1 by increasing the observation time 2τ. This agrees qualitatively with the earlier result in [38].

The phenomenological model of the spectral diffusion describes correctly also the manifestation of the spectral diffusion in a CPMG experiment. Indeed, as has been shown in [20] the electron spin decoherence in this case slows down according to Equation 7 and Equation 8. The decay of the CPMG echoes with *n* >> 1 occurs slower than for the primary echo if one compares the echo amplitudes with the same total time interval of the observation, i.e., the time 2τ in the primary echo experiment should be equal to the time 2*n*τ in the CPMG experiment.

**Figure 10:**
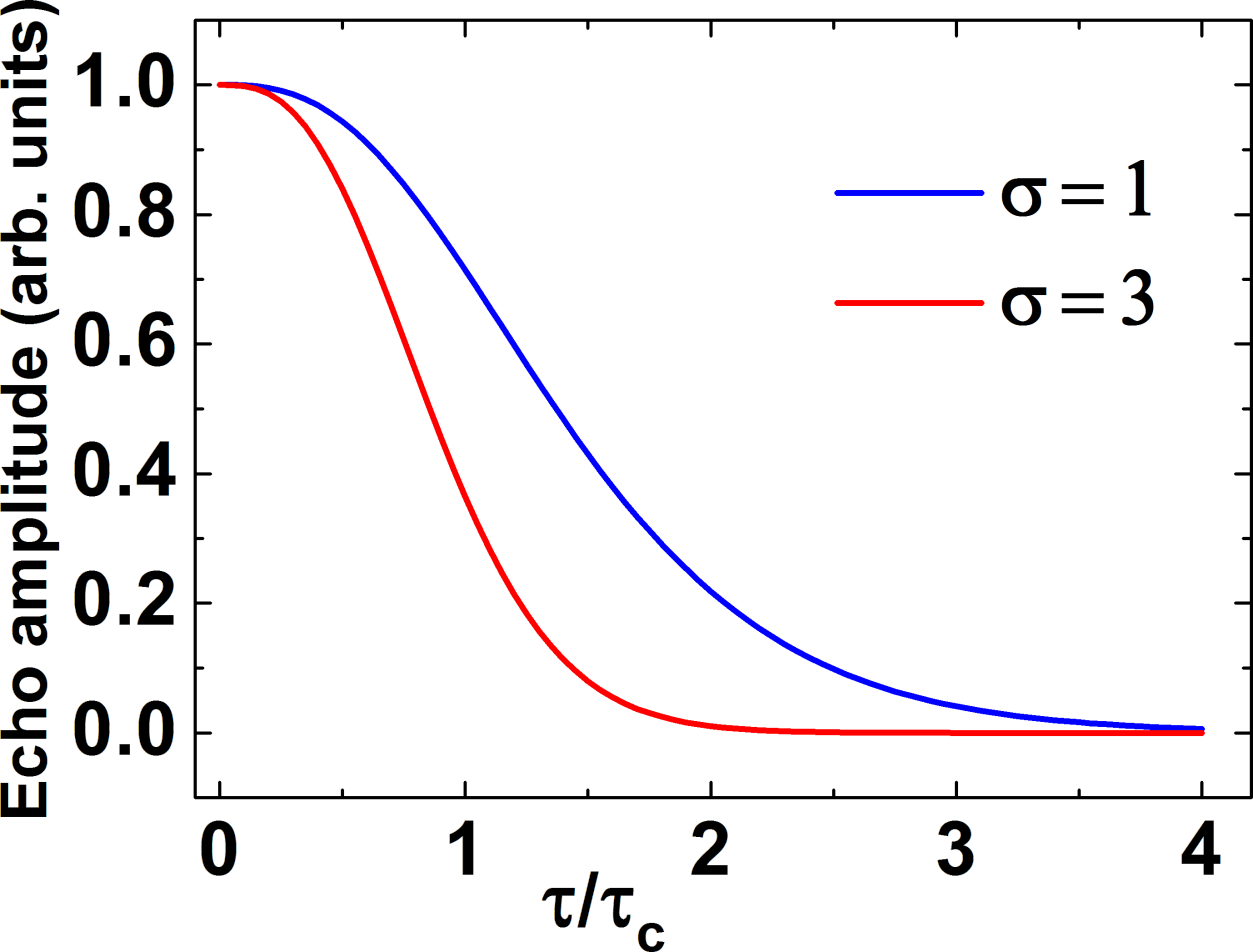
The calculated decay of the primary echo signal as a function of the time delay τ between the two pulses. τ is measured in units of the correlation time τ_c_ for the normal stochastic process of the spectral diffusion and the dispersion of the resonance frequency σ is given in units of 1/τ_c_^2^. The calculations were made for the two values of the dispersion σ = 1 (blue) and σ = 3 (red).

**Figure 11:**
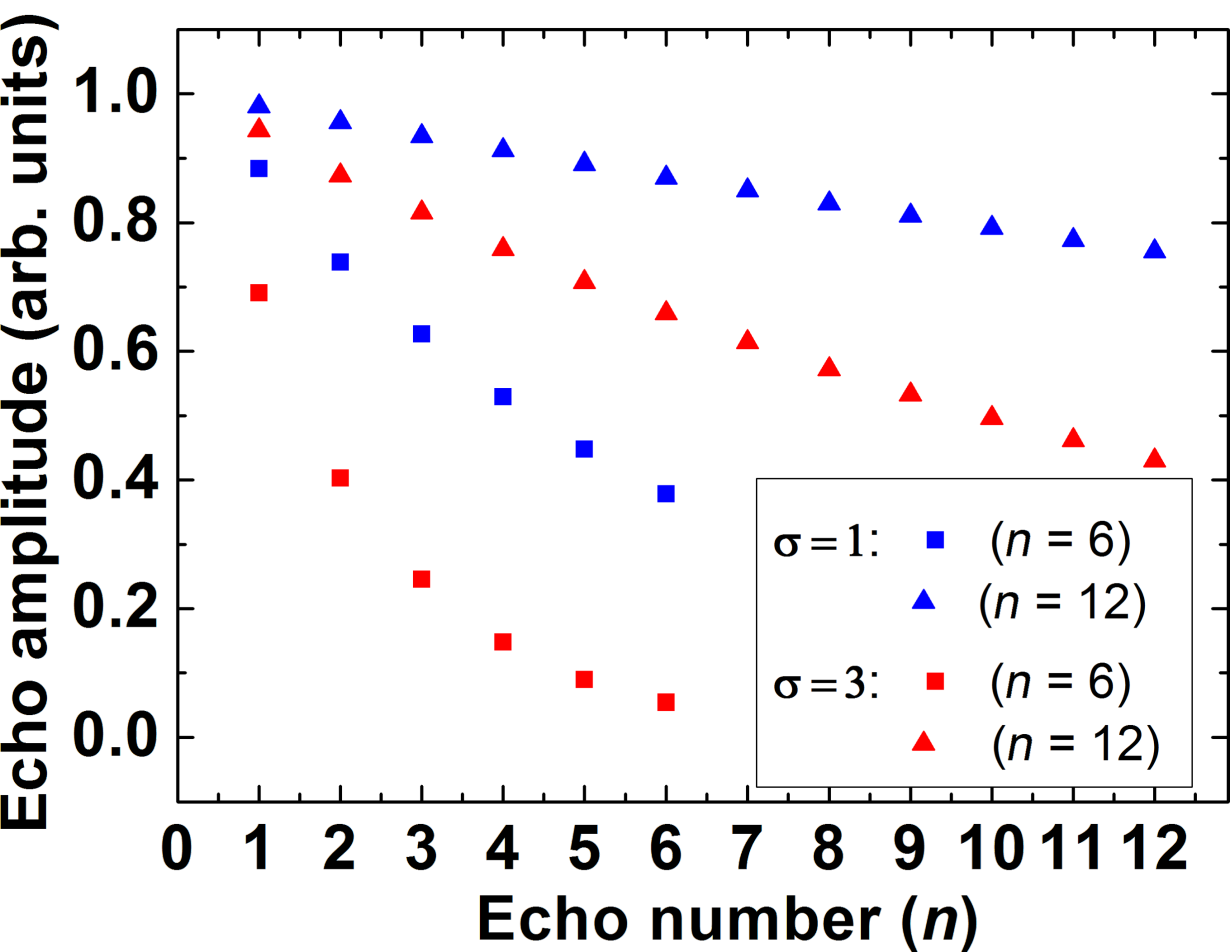
The calculated decay of the echo signal in the CPMG experiment as a function of the number *n* of the π-pulses in the CPMG protocol. The results of the modeling are presented for the same values of the dispersion σ as in Figure 10, σ = 1 (blue symbols) and σ = 3 (red symbols). The calculations were made for *n* = 6 (squares) and *n* = 12 (triangles). The total observation time of 10τ_c_ is the same as in Figure 10.

The decay of the primary echo signal and the decay of the echo signal in the CPMG pulse protocol with 6 and 12 π-pulses calculated with Equation 7 and Equation 8 are shown in Figure 10 and Figure 11, respectively. In numerical calculations the time τ is given in units of the correlation time τ_c_ for the normal stochastic process of the spectral diffusion and the dispersion of the resonance frequency σ is given in units of 1/τ_c_^2^. In Figure 11 the decay of the CPMG echo signal is plotted as a function of the number *n* of the CPMG echo. Obviously, Figure 10 demonstrates a rather complicated kinetics of the decay of the signal of the primary echo due to the spectral diffusion (see also Equations 9, 10 and 11). As can be seen there, with increasing the dispersion the contribution of the spectral diffusion to the spin decoherence increases and the echo signal decays faster. It should be noted that for sufficiently large time intervals between the pulses the decay of this signal can be described by the simple exponent (see, Equation 10) albeit this simple dependence holds only for the tail of the spin echo signal decay (Figure 10).

From these calculations the following conclusions can be drawn. Similar to the situation with the primary echo the intensity of the CPMG echo signals decays faster with increasing the dispersion σ (cf. squares and triangles in Figure 11). The dependence of the decay of the intensity of the CPMG echo as a function of its number *n* can be described by a simple exponent, in contrast to a more complicated behavior in the case of the primary echo. Note that the total observation time in Figure 10 and Figure 11 is equal and amounts to10τ_c_. From the point of view of potential application of electron spins as qubits, the slowing down of the spin decoherence as compared to the primary echo is certainly an interesting effect. Furthermore, for a given time interval of the observation the slowing down of the spin decoherence increases with the increase of the number of refocusing π-pulses in the CPMG protocol.

**Figure 12:**
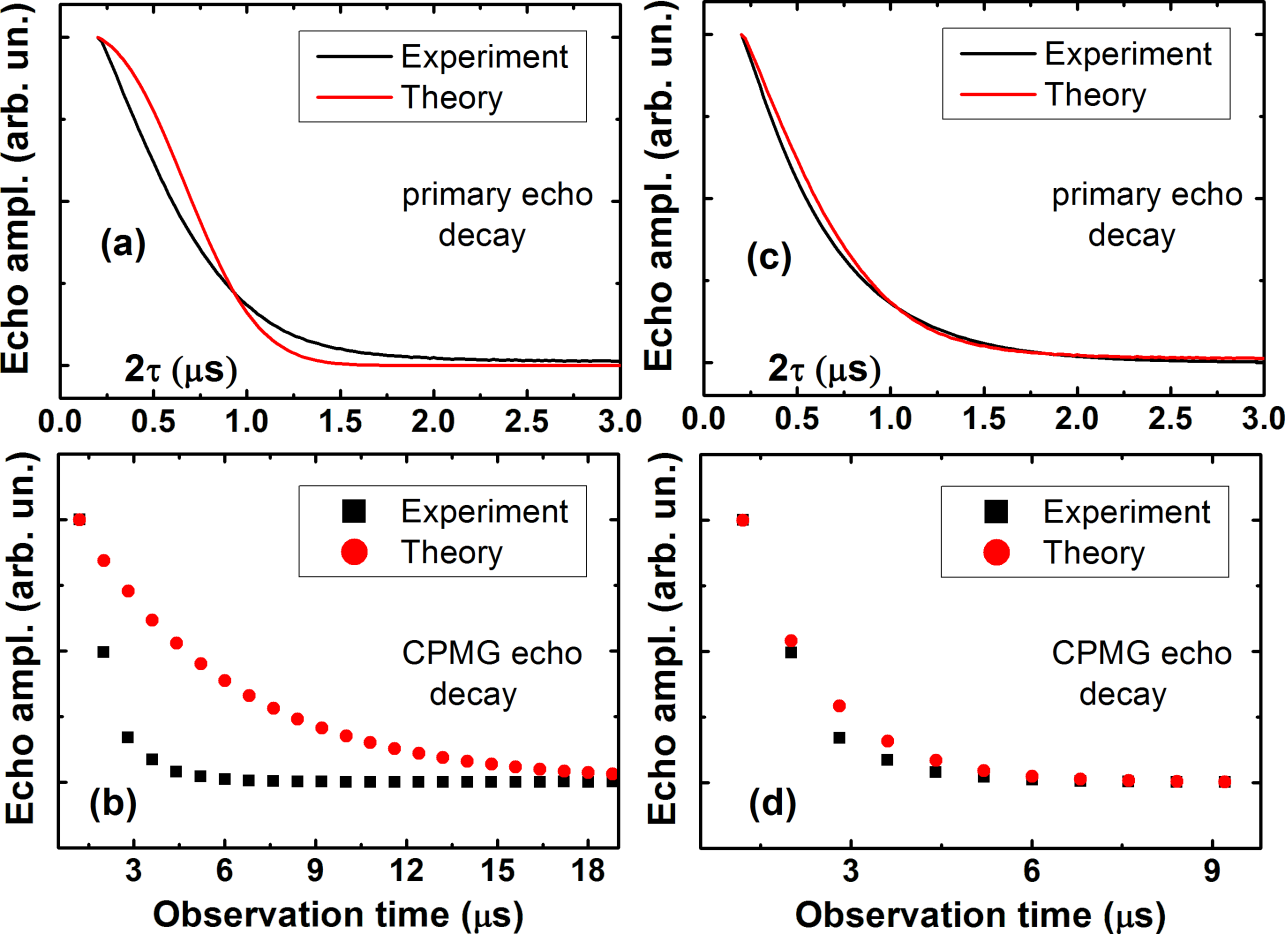
Comparison of the experimental and model dependences of the decay of the primary (a,c) and CPMG echoes (b,d) for complex **P1** at *T* = 80 K. The model curves in (a,b) were calculated according to Equation 7 with the correlation time τ_c_ = 2 µs, the diffusion barrier *d* = 0.7 nm, and στ_c_^2^ ~ 32, and in (c,d) with τ_c_ = 120 ns, *d* = 0.68 nm, and στ_c_^2^ ~ 0.13, respectively.

Thus the calculations presented above allow one to interpret qualitatively a drastic difference between the experimentally observed fast decay of the primary echo signal and the much slower decay of the CPMG echoes as shown in Figure 3 which demonstrates the efficiency of the CPMG protocol for the elimination of the effect of spectral diffusion on the electron spin decoherence in the studied molecular complexes. We have attempted to fit experimentally observed kinetic curves of the echo signal decay using Equation 7. Two examples with different fit parameters are presented in Figure 12a,b and Figure 12c,d, respectively.

The results shown in Figure 12 demonstrate that by varying parameters of the theoretical model, namely, the dispersion of the frequency σ and the frequency correlation time τ_c_ one can, in principle, fit the experimental data using Equation 7 reasonably well (see Figure 12c,d). However, such a good agreement with experiment is achieved by using a rather unrealistic value of τ_c_ = 120 ns. This correlation time is at least an order of magnitude smaller than that expected for a mutual flip-flop process responsible for the nuclear spin diffusion. Taking a more realistic value of τ_c_ = 2000 ns worsens the agreement substantially (see Figure 12a,b). This implies that although the theoretical model described above can provide a reasonably good qualitative description of the experimentally observed echo decay kinetic curves, but, on the quantitative level, in addition to the spin diffusion one has to take into account also other sources of stochastic modulation of the HF interaction.

Indeed, there are also other experimental observations which indicate that the nuclear spin diffusion is not only one source of a random modulation of the HF interaction in the studied materials. It should be noted that the electron spin dephasing time *T*_m_ obtained both with the primary echo and the CPMG pulse protocol is generally longer at the Q-band than at the X-band (Figure 4 and Figure 6). At both ESR frequencies the *T*_m_ time is systematically shorter for complex **P2** as compared to complex **P1** due to a larger number of magnetic nuclei (^14^N and ^1^H) in the former complex as compared to the latter one. The difference of the decoherence rate in the X- and Q-band measurements possibly indicates that the stochastic modulation of the HF interaction is caused not only by the nuclear spin diffusion but also by the stochastic changes of the nuclear spin projections due to the nuclear spin-lattice relaxation. Since the latter slows down with increasing the field strength [39], its possible effect on *T*_m_ should decrease too, which could qualitatively explain the difference of the electron spin dephasing rates in the two ESR frequency bands. Furthermore, the rate of the nuclear mutual flip-flops may be magnetic field dependent. In particular, the field-induced inhomogeneous broadening of the NMR line could make flip-flop processes less efficient. In any case, on the quantitative level, this interesting problem requires a special theoretical treatment to be addressed separately.

There is one more experimental observation which indicates a possible contribution of the nuclear spin-lattice relaxation to the electron spin dephasing induced by the HF interaction. Indeed, the spectral diffusion induced by random changes of the HF interaction caused by the nuclear spin diffusion is expected to be independent of temperature. Experimentally, the electron spin dephasing time *T*_m_ decreases with increasing temperature (Figure 4). This suggests additional temperature dependent contributions to the stochastic modulation of the HF interaction in the studied complexes. Such contributions, which could explain the observed temperature dependence of *T*_m_, may arise due to a random modulation of the HF interaction by a temperature dependent nuclear spin-lattice relaxation and/or by molecular mobility, e.g., by rotation of the CH_3_, CH_2_CH_3_ or other groups. Arguably, at low temperatures the local HF magnetic field acting on the electron spin is most effectively modulated by the nuclear spin diffusion. With increasing temperature a contribution of the nuclear spin-lattice relaxation to the stochastic modulation of the HF interaction, i.e., to the spectral diffusion, may become significant. As has been shown in [30,33], these two spectral diffusion mechanisms lead to different kinetics of the electron spin phase relaxation. The difference originates from the fact that in the case of the nuclear spin diffusion in an elementary act two spins are involved into the flip-flop process, while in the case of the spin-lattice relaxation each nuclear spin flips (or flops) independently. It should be noted that there are further significant differences between the two above discussed mechanisms. In the case of the nuclear spin diffusion its contribution to the electron spin dephasing is determined by the HF coupling to the nuclear spins at distances larger that the diffusion barrier. In the case of the nuclear spin relaxation all nuclei, including those inside the diffusion barrier, contribute to the shortening of *T*_m_. In addition, also the nuclear spin-lattice relaxation time depends on the distance between a particular nucleus and the paramagnetic center. This circumstance, as well freezing of the motion of the propyl groups may be responsible for the reduction of the stretching exponent *b* < 1 of the spin echo decay of complex **P2**.

Finally, we note on the specifics of the use of the CPMG pulse sequences in an ESR experiment. As it was originally pointed out in [40], unlike in an NMR experiment on non-magnetic substances where the first pulse excites the complete absorption line of the nuclear spins under study, it is often not the case for the inhomogeneously broadened ESR line. Due to a selective excitation of this line, the electron spins are turned by the first microwave pulse by different angles depending on their offset from the resonance frequency. As a result, in addition to the CPMG echoes which are basically the refocused primary echo, other unwanted echoes appear. Complications due to a selective excitation of spins in CPMG ESR experiments were later observed in several works [41–45]. However, up to now a detailed analysis of the underlying mechanisms giving rise to these complications, their impact on the determination of the spin dephasing time and the ways of elimination of unwanted effects were not elaborated sufficiently. The phase cycling which we use in our work as well as other cycling protocols [44] are definitely helpful in improving the quality of a CPMG ESR experiment. A more detailed theoretical and experimental treatment of this very interesting problem will be published elsewhere.

## Conclusion

We have experimentally studied the dephasing time *T*_m_ of Cu(II) spins in single crystals of diamagnetically diluted mononuclear [*n*-Bu_4_N]_2_[Cu(opba)] (1%) in the host lattice of [*n*-Bu_4_N]_2_[Ni(opba)] (**P1**) and of diamagnetically diluted mononuclear [*n*-Bu_4_N]_2_[Cu(opbo*n*-Pr_2_)] (1%) in the host lattice of [*n*-Bu_4_N]_2_[Ni(opbo*n*-Pr_2_)] (**P2**) by pulse ESR measurements at X- and Q-band frequencies. We have found that application of the special Carr–Purcell–Meiboom–Gill (CPMG) pulse protocol significantly increase the *T*_m_ time of both complexes as compared to the results of the standard two-pulse primary echo measurements. Our theoretical analysis shows that this effect is related to an efficient suppression by the CPMG multi-pulse sequence of the detrimental influence on the spin coherence lifetime of the spectral diffusion induced by the stochastic modulation of the HF interaction. This stochastic modulation can be caused by several random processes such as the nuclear spin diffusion, the nuclear spin-lattice relaxation, molecular mobility, e.g., the random rotation of CH_3_, CH_2_CH_3_ groups, etc. At low temperatures the first mechanism is dominating whereas with increasing temperature the other two may become relevant, thus explaining the experimentally observed decrease of the electron spin dephasing time *T*_m_. The systematically shorter *T*_m_ times at all temperatures found for complex **P2** as compared to **P1** can be obviously related to a larger number of magnetic nuclei in the former complex that additionally contribute to the spectral diffusion mechanism of the electron spin dephasing. It is likely for this reason the *T*_m_ times of both complexes measured by the primary spin echo appear shorter as in a number of other copper, vanadyl and chromium complexes reported and discussed in recent literature in the context of quantum information processing (see, e.g., [23,45–48]). This has to be kept in mind while considering possible applications of Cu(II)–(bis)oxamato and Cu(II)–(bis)oxamidato complexes in molecular electronic devices. Our experimental results show that this drawback can be to a large extent overcome by application of the multi-pulse CPMG sequences. Additionally, this approach offers a possibility to assess if some mechanism of the spectral diffusion affects the electron spin coherence by measuring the *T*_m_ time with primary and CPMG echoes which in this case should be substantially different. What specific mechanism of the spectral diffusion is active can be concluded from the analysis of the kinetics of the phase relaxation and its dependence on temperature, HF interaction, etc.

Finally, on the experimental level, we have suggested a modification of the standard CPMG pulse protocol that enabled an effective elimination of additional unwanted echoes arising due to a selective excitation of electron spins.
